# Transcriptomic Characterization of Hepatocellular Carcinoma with *CTNNB1* Mutation

**DOI:** 10.1371/journal.pone.0095307

**Published:** 2014-05-05

**Authors:** Xiao Ding, Yuan Yang, Baoda Han, Chengzhi Du, Naiqing Xu, Huanwei Huang, Tao Cai, Aiqun Zhang, Ze-Guang Han, Weiping Zhou, Liang Chen

**Affiliations:** 1 National Institute of Biological Sciences, Beijing, Beijing, China; 2 Eastern Hepatobiliary Surgery Hospital, Yangpu, Shanghai, China; 3 The General Hospital of People's Liberation Army (301 Hospital), Beijing, China; 4 Shanghai Center for Systems Biomedicine, Shanghai Jiao Tong University, Shanghai, China; 5 Collaborative Innovation Center of Cancer Medicine, National Institute of Biological Sciences, Beijing, Beijing, China; Fudan University, China

## Abstract

**Purpose:**

Hepatocellular carcinoma (HCC) is the sixth most common solid tumor worldwide and the third leading cause of cancer-related death. HCC is a particularly serious threat to the Chinese population. Although many molecular alterations are known to be involved in the tumorigenesis of hepatocytes, no systemic survey has examined the somatic mutations in HCC samples from Chinese patients. Our goal was to elucidate somatic mutations in Chinese HCC patients and investigate the possible molecular mechanisms involved in tumorigenesis.

**Experimental Design:**

A total of 110 hepatitis B virus (HBV)-positive HCC samples and 46 HBV-negative HCC samples were genotyped for hot-spot mutations in the *CSF1R*, *CTNNB1*, *KRAS*, *BRAF*, *NRAS*, *ERBB2*, *MET*, *PIK3CA*, *JAK1*, and *SMO* genes. The transcriptomes of the *CTNNB1* mutation-positive HCC samples from the HBV-positive patients (CB+ HCC) were compared to adjacent non-cancerous livers, and significantly altered genes were functionally validated *in vitro*.

**Results:**

*CTNNB1* mutations accounted for the majority of the mutations detected in our study. A slightly higher mutation rate was found in the HBV-positive patients than in their negative counterparts. A distinct pattern of *CTNNB1* mutation was detected in these two populations, and drastic changes at the transcriptomic level were detected in the CB+ tumors compared to adjacent non-cancerous livers. Potential tumor suppressors (FoxA3 and Onecut1) and oncogenes (MAFG and SSX1) were functionally validated.

**Conclusions:**

Our work is the first systemic characterization of oncogenic mutations in HCC samples from Chinese patients. Targeting the Wnt-β-catenin pathway may represent a valid treatment option for Chinese HCC patients. Our work also suggests that targeting ONECUT1, FOXA3, SSX1, and MAFG may be a valid treatment option for CTNNB1 mutation positive HCC patients.

## Introduction

Hepatocellular carcinoma (HCC) is the most common liver cancer and the third leading cause of cancer death, with a annual incidence of approximately 600,000 people worldwide [1], [2]. Although significant efforts have been made to control the incidence and mortality of HCC, a recent survey conducted in the USA revealed that the age-adjusted HCC incidence rates tripled between 1975 and 2005 [3], ranking it the highest in average annual percent increase of the top 15 cancers according to incidence in the USA. Despite the progress in diagnosis and therapy, the prognosis of HCC remains dismal.

HCC tumorigenesis is highly complex and still not completely understood. Many molecular alterations have been reported in HCC: p53, Rb/p16, PTEN, RUNX3, RAS family proteins, such developmental pathways as the Wnt pathway and Hedgehog pathway, growth factors and their receptors, and telomerase have all been shown to be altered in HCC [4], [5]. All of these alterations fit the popular view that a histocyte must go through “more than two hits” before malignant transformation; these hits often include a gain-of-function mutation in a proto-oncogene and/or a loss-of-function mutation in a tumor suppressor gene [6]. Oncogenes are thought to play a dominant role in this process.

Liver cancer poses a significant threat to the Chinese population, claiming 400,000 lives each year, second to only lung cancer (Statistics for the year of 2004 released by Ministry of Health of China in 2007), and the incidence of HCC is particularly high in some regions of China. Despite this serious threat, no systemic etiological survey of oncogenic mutations in HCC has been performed in the Chinese population. In this study, we surveyed mutations in the oncogenes known to be important in liver cancer and found that *CTNNB1* is the predominantly mutated oncogene in Chinese HCC patients. We report that distinct patterns of mutation exist in hepatitis B virus (HBV)-negative and HBV-positive populations, and dramatic differences were found in the expression and function of signaling and metabolic pathways in *CTNNB1* mutation-positive HCC samples from the HBV-positive patients (CB+ HCC) compared to para-tumoral tissues at the transcriptomic level. We also highlight some potential coordinating tumor suppressors and oncogenes for the *CTNNB1* mutant tumors. Our work represents the first report of a comprehensive molecular etiological survey of HCC in the Chinese population.

## Materials and Methods

### 1. Ethics Statement

This study was approved by Shanghai Eastern Hepatobiliary Surgery Hospital, Shanghai, China, and the Institutional Committee at the National Institute of Biological Sciences, Beijing (NIBS). Written consent was obtained from every patient who donated tissues.

### 2. Specimen Collection

All cases were reviewed by pathologists to confirm the stage of liver cancer (TNM system and BCLC system), tumor histology, and tumor content. The criteria for resection included the absence of distant metastasis or main portal vein thrombosis, an anatomically resectable disease, and an adequate liver function reserve, as assessed by liver biochemistry and indocyanine green retention after 15 minutes. None of the patients had received other therapies, including chemo-embolization or chemotherapy, before the tumor was resected. Serum HBsAg (hepatitis B virus surface antigen) and anti-HBs (hepatitis B virus surface antibody) levels were assayed using an enzyme immunoassay test (Abbott Laboratories, Chicago, IL, USA). A portion of each fresh sample was treated with the RNAlater reagent (Invitrogen, Carlsbad, CA, USA) overnight at 4°C and was stored at -80°C until use; the remaining tissue samples were immediately snap-frozen in liquid nitrogen after resection and store at -80°C.

### 3. Mutational analysis

Genomic DNA was extracted from the HCC tissues using the Tiangen DNA Mini Kit (Tiangen, Beijing, China), and mutation sites with the highest frequencies were selected according to the COSMIC website (http://www.sanger.ac.uk/genetics/CGP/cosmic/). Single-nucleotide polymorphisms (SNPs) in the *CSF1R*, *CTNNB1*, *KRAS*, *BRAF*, *NRAS*, *ERBB2*, *MET*, *PIK3CA*, *JAK1*, and *SMO* genes with frequencies higher than 0.1% mutation in HCC (Table S2) were evaluated in all of the DNA samples using the SequenomMASSARRAY Service (CapitalBio, Beijing, China).

### 4. RNA-sequencing (RNA-seq) and Gene-Ontology analysis

Among the HCC samples, four paired samples derived from HBV-positive patients that harbored the *CTNNB1* mutation were selected for RNA-seq. Total RNA was extracted from these samples using Trizol (Invitrogen, Carlsbad, CA). The library was constructed by the Sequencing Center at NIBS. RNA single-end sequencing was performed using an Illumina GAII analyzer.

For the data analysis, 45-bp sequences called by the Illumina pipeline were mapped to the human genome (hg19) using Tophat (v2.0.4). Gene annotation and the calculation of FPKM (fragments per kilobase of transcript per million mapped reads) values were performed using Cufflinks (v2.0.2), with the provision of a GTF annotation file (hg19).

We have deposited our RNA-seq data in Gene expression omnibus, NCBI (accession number: 5048). It's now publicly available online with the following link: http://www.ncbi.nlm.nih.gov/geo/query/acc.cgi?token=wdujaqwefhmdpcx&acc=GSE55048.

Differences in gene expression were assessed using Cuffdiff, with a false discovery rate correction for multiple testing. Genes with p-values ≤0.05 and a fold change ≥2 were considered differentially expressed.

To identify the overrepresented biological categories within each cluster, a Gene Ontology (GO) term analysis was applied. The percentage of the genes within each category in relation to the total amount of genes in each cluster was calculated, and hierarchical clustering was conducted to group the clusters according to their similarities in gene function representation.

### 5. Dual luciferase reporter assay

M50 Super 8x TOPFlash reporter plasmid[7] (kindly gifted by Dr. Wei Wu) and mutant β-catenin (T41A) plasmids were transfected into 293T cells in 96-well plates using the Vigofect reagent (Vigorous Biotechnology, Beijing, China) according to the supplier's recommendations. The cells were harvested 36–48 h post-transfection. Reporter assays were performed using the Dual-Luciferase Reporter Assay with internal control of Renilla lucicerase plasmid (Promega, Madison, WI, USA, kindly gifted by Dr. Wei Wu) according to the manufacturer's protocol.

### 6. Cell growth assay for potential tumor suppressors and oncogenes

We selected 35 potential tumor suppressors from the list of downregulated genes in the RNA-seq dataset and cloned these genes into the pLIP lentiviral vector that contained a FLAG tag. Each vector was transfected into 293T cells, and a western blot analysis was used to verify the expression of the construct with anti-FLAG antibody (Sigma-Aldrich Co. LLC, St. Louis, MO USA). Lentiviruses were packaged in 293T cells following cotransfection with the pMD2.G and psPAX2 vectors, and the virus was stored at −80°C.

To determine the potential tumor-suppressive functions of these genes, we selected three cell lines, including HepG2, Huh7, Hep3B, from ATCC (Manassas, VA, USA). All of the cells were grown in 384-well plates and were infected with 50-µl lentivirus encoding the potential tumor suppressor at 18 h after the cells had adhered to the dish. The virus was removed at 12 h post-infection, and the cells were allowed to grow for another 5 days. The relative cell number was detected using Cell Titer Glo (Promega, Madison, WI, USA) according to the manufacturer's protocol. Infection of each gene was repeated at least three times in triplicate form each time.

### 7. Statistics

A Kaplan–Meier survival curve analysis was performed using the Prism software. For comparing effects of transcription factors on cell growth, student's t-tests were calculated with Excel version 2007 software (Microsoft, Redmond, WA, USA) using an unpaired two-tailed analysis. A P-value <0.05 (*) was considered statistically significant. A P-value <0.01 (**) was considered very statistically significant.

## Results

### 1. Tumor sample collection

**Figure 1:**
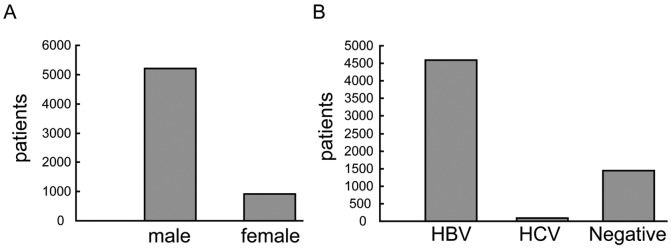
Characteristics of the HCC patients. A total of 6121 patients were diagnosed with HCC in our hospital during the past four years. A: The male to female ratio for this cohort (5203 males and 918 females). B: The majority of the patients (4590; 74.99%) were positive for HBsAg, whereas 83 patients (1.36%) were positive for HCV in this cohort.

From Jan 2009 to May 2013, 6121 patients who underwent partial hepatectomy at Eastern Hepatobiliary Surgery Hospital, Shanghai, China, were diagnosed with HCC by a post-operative pathological examination. A total of 5203 males and 918 females were diagnosed with HCC (Fig. 1A). Among them, 4590 patients (74.99%) were positive for HBsAg, and 83 patients (1.36%) were positive for hepatitis C virus (HCV) (Fig. 1B). Our data are consistent with earlier reports showing that HBV is highly associated with HCC in the Chinese population and that HCC predominantly affects males [8], [9]. However, a 5.67∶1 male:female ratio was observed in this study, which was slightly higher than that in earlier reports [8], [10].

To compare the frequency of oncogenic mutations in the HBV-positive and HBV-negative patients, we consecutively collected a total of 110 HBV-positive HCC samples and 46 HBV-negative HCC samples in our study. The average age of the patients was 53.09; specifically, the average age for the HBV-positive patients was 52.09, and the average age for the HBV-negative patients was 55.48. Cases were included in this study based on the following criteria: a review confirming the pathological diagnosis of HCC; a tumor specimen containing a minimum of 70% tumor cells; sufficient tissue available for a comprehensive analysis; and corresponding normal tissue also available for analysis. The detailed characteristics of the patients are listed in Table 1, and the information for all of the patients is listed in Table S1.

**Table 1:** Characteristics of HCC patients.

Characteristic		Total	HBsAg(+)	HBsAg(-)
No.of patients		156	110	46
Age(years)		53.09	52.09	55.48
SD		11.19	10.95	11.50
Clinical stage(TNM system)				
T1N0M0		58	42	16
T2N0M0		49	36	13
T3N0M0		41	25	16
T3N1M1		1	1	0
T4N0M0		7	6	1
Clinical stage(BCLC system)				
A		31	24	7
B		96	64	32
C		27	20	7
D		2	2	0
HCC differentiation grade				
Grade2		22	18	4
Grade3		119	82	37
Grade4		3	3	0
Mix	Grade1+2	1	0	1
	Grade2+3	9	5	4
	Grade3+4	2	2	0

### 2. *CTNNB1* mutations are the predominant somatic mutations detected in Chinese HCC samples

Genetic events that lead to the activation of oncogenic signaling pathways are thought to play dominant roles in the process of transformation of a histocyte, and the elucidation of these events will ultimately lead to the development of targeted therapies. HCC is the second leading cause of cancer-related death in the Chinese population; however, no systemic survey of somatic mutations has been performed in HCC patients of Chinese ethnicity. Therefore, we chose to investigate mutations in known oncogenes in HCC samples from Chinese patients.

**Figure 2:**
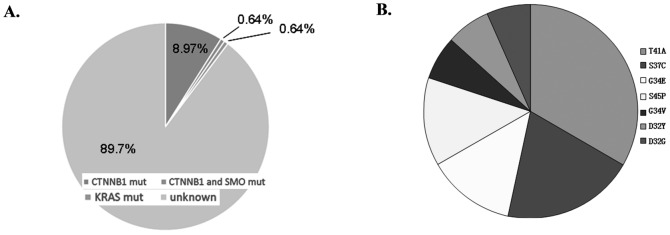
Statistical analysis of mutational information in the Chinese HCC patients. A: *CTNNB1* mutation is the predominant mutagenic event. Of the 156 samples assessed, 15 cases (9.6%) were found to have *CTNNB1* mutations. 14 cases (8.97%) had CTNNB1 mutation only, one case (0.64%) harbored CTNNB1 mutation and SMO (K575M) mutation, and one case (0.64%) had a KRAS (G12D) mutation. B: All of the mutations in CTNNB1 were clustered within the N-terminal region; five cases exhibited the T41A mutation, whereas three cases had the S37C mutation. The G34E and S45P mutations were found in two cases each, and the G34V, D32Y and D32G mutations were detected in only one case each in this study.

Due to the lack of systemic mutational oncogene information for the Chinese HCC population, we choose to genotype the frequently mutated proto-oncogenes that are listed in the Catalogue Of Somatic Mutations In Cancer (http://cancer.sanger.ac.uk/cancergenome/projects/cosmic/). Ten genes, including *CSF1R*, *KRAS*, *BRAF*, *NRAS*, *ERBB2*, *MET*, *PIK3CA*, *JAK1*, *SMO*, and *CTNNB1*, rank top among this list. A total of 33 known mutational sites for the 10 proto-oncogenes were checked in 156 HCC patients (Table S2). Interestingly, of the 156 samples evaluated, 15 cases displayed *CTNNB1* mutations (9.6%), one case had a *KRAS* (G12D) mutation, and one case exhibited a *SMO* (K575M) mutation (Fig. 2A). A slightly lower mutation rate was observed in the HBV-negative HCC samples (6.5%) than the HBV-positive samples (10.9%). All of the mutations in *CTNNB1* clustered within the N-terminal region. Five cases exhibited the T41A mutation, and three cases of the S37C mutation were observed. The G34E and S45P mutations were found in two cases each, and the G34V, D32Y, and D32G mutations were found in only one case each (Fig. 2b). Interestingly, the *SMO* mutation coexisted with the *CTNNB1* T41A mutation in an HCC sample from an HBV-positive patient. We also observed a difference in the mutational pattern of *CTNNB1* in the HBV-negative and HBV-positive populations. HBV-negative HCC predominantly harbored the G34 mutations (one case each of G34V and G34E and one case of S45P), whereas mutation at this site was observed only in one HBV-positive patient (one case of G34E). In contrast, *CTNNB1* mutations in the HBV-positive HCC samples were clustered at phosphorylation sites, such as S37 (three cases), T41 (five cases), and S45 (one case). The detailed information concerning these mutations is listed in Table 2.

**Table 2:** Information of β-catenin mutation detected in Chinese HCC patients.

Patients ID	SNP site	Amino acid change	HBsAg	Other coordinated mutation
082552	121A>G	T41A	+	
076706	110C>G	S37C	+	
080049	121A>G	T41A	+	
070830	101G>A	G34E	+	
068345	101G>A	G34E	−	
070220	133T>C	S45P	+	
078222	110C>G	S37C	+	
078265	95A>G	D32G	+	
080019	121A>G	T41A	+	
079834	110C>G	S37C	+	
074894	94G>T	D32Y	+	
076994	121A>G	T41A	+	
079032	133T>C	S45P	−	
080280	101G>T	G34V	−	
081224	121A>G	T41A	+	SMO_1724A>T

### 3. Transcriptomic characterization of the HCC samples harboring *CTNNB1* mutations

Since CTNNB1 is the predominantly mutated proto-oncogene in HCC samples, we therefore tested whether β-catenin played an important role in growth and survival for HCC cells. Using two siRNAs of different knocking-down efficiency (Fig S1A), we found that both siRNAs reduced growth rate of HepG2 cells, an HCC cell line harboring mutant β-catenin (Fig S1B). Our result is consistent with recent observation by Monga and colleagues that β-catenin plays an important role in proliferation, survival and viability of HCC cells [11]. Indeed, *CTNNB1* is an important oncogene in gastrointestinal tumors, and this gene is tightly regulated during normal liver development [12]. The functional deregulation of β-catenin has been associated with HCC incidence, and deletion of the adenomatosis polyposis coli (APC) gene and subsequent activation of the Wnt pathway results in hepatocarcinogenesis in a mouse model [13].

Given that *CTNNB1* was the predominantly mutated gene detected in our study and that a majority of HCC patients are HBV positive, we then investigated abnormalities in CB+ HCCs. To this end, we took advantage of the RNA-seq next-generation sequencing technology to compare the transcriptome of the HCC samples to the adjacent non-cancerous livers derived from 4 patients.

**Figure 3:**
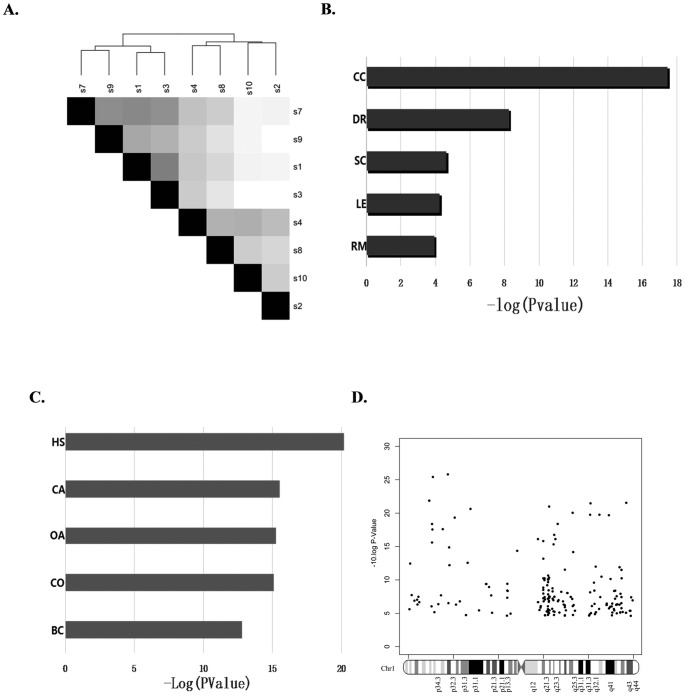
Transcriptomic analysis of the *CTNNB1* mutation-positive tumors compared to the para-tumoral tissues. A: Samples from the HBV-positive and *CTNNB1* mutation-positive (CB+) tumors were relatively homogenous. B: cell cycle (CC), DNA replication (DR), signature of small cell lung cancer signaling (SC), signature of systemic lupus erythematosus signaling (LE), and pyrimidine metabolism (RM) were the top five significantly upregulated functional pathways. Bar graph shows based on – log value of statistical significance. C: signature of hemostasis signaling (HS), carboxylic acid catabolic process (CA), organic acid catabolic process (OA), signature of coagulation signaling (CO), and signature of blood coagulation signaling (BC) are the top five down-regulated functional pathways. Bar graph shows based on – log value of statistical significance. D: Most of the upregulated genes were clustered on chromosome 1.

Interestingly, the average Pearson's correlation coefficient (PCC) (0.992075) for any two adjacent non-cancerous tissues, the average PCC (0.992699) for any two cancer tissues, and the average PCC (0.991389) for any cancer and non-cancerous tissue pair suggested that there was a high degree of biological homogeneity between the HCC tissues, between non-cancerous adjacent tissues, and between tumor and non-tumor tissues (Fig. 3A.). This result indicated that our strict selection standard for the CB+ HCC and para-tumoral tissues enabled us to analyze a relatively homogenous cohort of HCC samples from highly heterogeneous diseases. RNA samples from the four CB+ HCCs and noncancerous para-tumoral liver tissues were sequenced and compared. We identified a total of 146 genes that were significantly upregulated and a total of 215 genes that were significantly downregulated in all of the CB+ HCCs (Table S3) compared to the para-tumoral tissues.

Interestingly, although we found typical Wnt pathway target genes, such as Axin2 and Myc, among the upregulated genes, the majority of the genes identified were not directly associated with the Wnt pathway or β-catenin function, suggesting that a global alteration in functionality had occurred in the tumor cells compared to the para-tumoral tissues. Therefore, we functionally grouped the upregulated and downregulated genes, with the pathway analysis revealing that cell cycle, DNA replication, signature of small cell lung cancer signaling, signature of systemic lupus erythematosus signaling, and pyrimidine metabolism were the top five significantly upregulated functional clusters (Fig. 3B). Conversely, signature of hemostasis signaling, carboxylic acid catabolic process, organic acid catabolic process, signature of coagulation signaling, and signature of blood coagulation signaling were the top five functional clusters that were downregulated (Fig. 3C). Additionally, we found that most of the upregulated genes were clustered on chromosome 1 (Fig. 3D).

### 4. Alterations in potential tumor suppressors and oncogenes that coordinate with β-catenin to transform hepatocytes

Although the *CTNNB1* mutation represents an important event leading to the tumorigenesis of hepatocytes, a mouse model overexpressing the liver-cancer associated β-catenin mutant showed that β-catenin itself was not sufficient to induce HCC [14]. Therefore, we attempted to identify possible molecular events that may coordinate with the β-catenin mutant during transformation.

**Figure 4:**
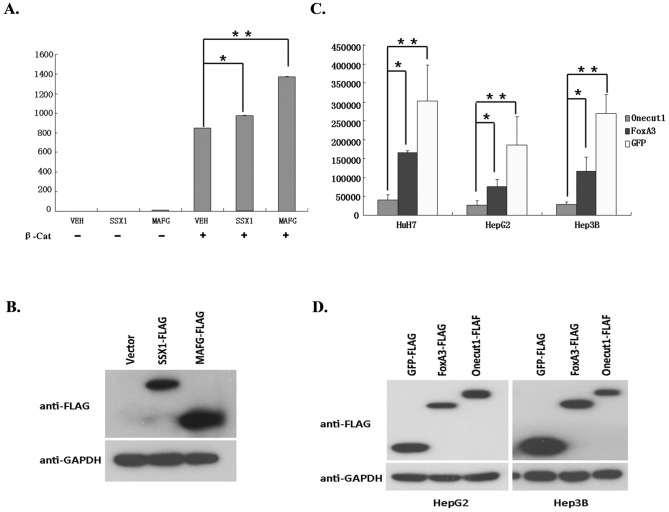
Potential tumor suppressors and oncogenes are involved in tumorigenesis. A: MAFG and SSX1 synergize mutant β-catenin on transcriptional activity. 293T cells transfected with vehicle (VEH), expression vector for FLAG tagged SSX1, or FLAG tagged MAFG with/without mutant β-catenin. (−) for without and (+) for with mutant β-catenin using Dual-Luciferase Reporter Assay System. Fold-change of luciferase activity is grafted. B: Representative Western blot analysis was conducted with anti-FLAG antibody on cells transfected with β-catenin and VEH, SSX1, or MAFG. C: Onecut1 and FoxA3 overexpression inhibits the growth of HCC cancer cells. D: Representative Western blot analysis was conducted with anti-FLAG antibody on HepG2 and Hep3B cells transfected with FLAG tagged GFP, ONECUT1 and FOXA3.*P value 0.05-0.01; **P value <0.01

First, we examined the upregulated genes and wondered if there were many genes in this list that could potentially synergize with the transcriptional activity of β-catenin. To test this possibility, we assessed the transcription factors that were upregulated in a significant fraction of the *CTNNB1* mutation-positive tumors versus the para-tumoral tissues using the dual luciferase reporter assay. Surprisingly, we found that MAFG and SSX1 significantly synergized with the transcriptional activity of β-catenin (Fig. 4A for luciferase activity and Fig. 4B for western analysis of MAFG and SSX1 expression). To the best of our knowledge, neither of these 2 transcription factors has been associated with β-catenin transcription activity in earlier studies.

We also examined the downregulated genes, as these genes may function as tumor suppressors, and tested this possibility by overexpressing these genes in HCC cell lines. Surprisingly, we found that overexpression of Onecut1 and FoxA3 potently inhibited the growth of the *CTNNB1* mutation-positive (HepG2) cell line and the negative (Huh-7 and Hep3B) cell lines (Fig. 3C for cell growth rate and Fig. 4D for representative western analysis of Onecut1 and FoxA3 in HepG2 and Hep3B). FOXA3, which is important for hepatocyte differentiation and development, was consistently downregulated in a significant portion of the *CTNNB1*-mutation positive samples; Onecut1 was also downregulated in a portion of the *CTNNB1* mutation-positive tumors. Our results are consistent with a recent report that tissue master transcriptional factors function as potent tumor suppressors [15].

### 5. *CTNNB1* mutation is associated with longer survival times

**Figure 5:**
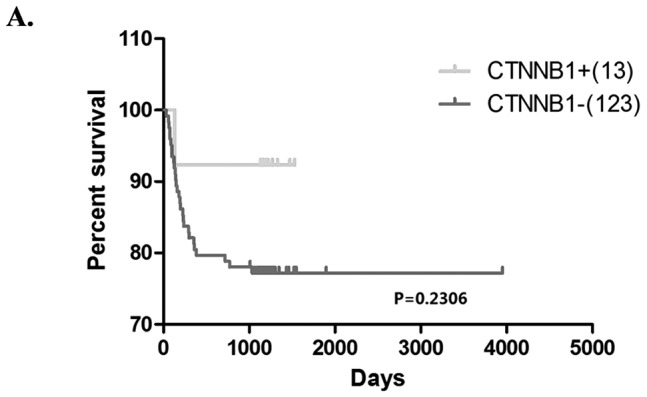
*CTNNB1* mutation-positive patients have a better prognosis. Statistical analysis of 110 HBV-positive patients and 46 HBV-negative HCC patients showed that the *CTNNB1* mutation-positive patients have a longer survival time; Difference was not significant.

Because an important issue for liver cancer patients is the poor prognosis of the disease, we compared the survival rate of the β-catenin mutation-positive and -negative populations. Consistent with earlier data[16], we found that the *CTNNB1* mutation-positive patients exhibited a non-significant trend of longer survival time than the negative patients (Fig. 5).

## Discussion

Liver cancer is the second most prevalent cancer in China. To the best of our knowledge, our current work represents the first comprehensive survey of mutated oncogenes in HCC tumors from patients of Chinese ethnicity. Our work revealed that mutations in *CTNNB1* are the predominant oncogenic alterations found in HCC cases in China. More importantly, we identified certain molecular events which may promote tumorigenesis in a *CTNNB1* mutation-positive tumor.

Different *CTNNB1* mutational frequency were reported in various studies [17] [18] [19] [16]. Our work further confirms that the frequency of CTNNB1 mutation of Chinese HCC patients fits in that of East Asian HCC population. Also, consistent with earlier work [16], *CTNNB1* mutation-positive patients were found to have a better prognosis than their *CTNNB1* mutation-negative counterparts.

Our observation that MAFG overexpression resulted in enhanced mutant β-catenin transcriptional activity can be explained by the following two possibilities: 1) MAFG can physically interact with β-catenin to affect its transcriptional activity; and 2) the transcribed target protein(s) interact with β-catenin to enhance its transcriptional activity. This observation is consistent with the idea that MAFG is a proto-oncogene. Similarly, SSX1 may exert its impact on β-catenin directly or indirectly. Regardless, it should be emphasized that we only evaluated the upregulated genes for their ability to synergize with β-catenin transcriptional activity. Other mechanisms, independent of synergizing with β-catenin transcriptional activity, remain to be determined. As neither protein has been functionally associated with β-catenin transcriptional activity, it is surprising to find that MAFG and SSX1 have the ability to synergize with the transcriptional activity of β-catenin. Therefore, our work deserves careful follow-up. Interestingly, we also found that MAFG and SSX1 were upregulated in the sample that contained both *CTNNB1* and *SMO* mutations, suggesting that these two genes are associated with β-catenin. Of note we observed that MAFG synergized with the transcriptional activity of both wild-type and mutant β-catenin. However, SSX1 synergized specifically with activity of mutant β-catenin (data not shown).

The downregulation of FoxA3 and Onecut1, which are important for hepatocyte differentiation and development, were observed in a portion of HCC samples. We validated that both had the potential to function as tumor suppressors. It is likely that these transcription factors may function in a manner similar to the recently reported lung transcription factor [15]. Interestingly, we found that FoxA3 was also downregulated in the *CTNNB1* mutation-positive HCC sample from an HBV-negative patient and in *CTNNB1* and *SMO* mutation-positive HCC, suggesting that the downregulation of this transcription factor is highly associated with mutations in *CTNNB1*.

We also found a higher β-catenin mutation rate in the HBV-positive patients, and the mutational pattern was different in the HBV-positive and HBV-negative patients. HBV-negative HCC predominantly harbors G34 mutations, whereas the mutations in the HBV-positive HCC samples were clustered at phosphorylation sites (S37, T41, and S45), suggesting that the tumorigenesis of these two subtypes could be different. Indeed, HBV contributes to tumorigenesis through its oncogenic components, mostly HBx [20], its integration, which can dysregulate such cellular genes as telomerase [21], [22], and the inflammation reaction during HBV infection [23]. This suggests that there are different underlying mechanisms of HCC tumorigenesis in HBV-positive and HBV-negative patients. Therefore, corresponding therapeutic strategies should be considered for these two patient subgroups.

In summary, our finding that β-catenin is the predominantly mutated oncogene in Chinese HCC patients suggests that targeting this pathway may be a viable option for the treatment of HCC patients in China. Indeed, efforts have been invested in developing reagents that inhibit β-catenin function, which could potentially lead to apoptosis in the cancer cells [24]. More broadly, components of the Wnt signaling pathway can be targeted during the treatment of HCC [25].

## Supporting Information

Figure S1 **knockdown of β-catenin reduced growth of Hep2G cell line.** A: Two siRNAs effectively knocked down protein level ofβ-catenin in HepG2 cell. B: β-catenin knocked down HepG2 cell showed significantly lower growth rate in comparison to that treated with control siRNA. HepG2 cells were transfected with human β-catenin (CTNNB1) siRNA or negative control siRNA. The cells were harvested at 48 and 72 hours post-transfection for western blotting and cell viability assay respectively. The siRNAs sequences used are listed below. siCat1: CCACAAGAUUACAAGAAACGGCUUU; siCat2: AAGUCCUGUAUGAGUGGGAAC; siCtr: AACAGUCGCGUUUGCGACUGG.
(TIF)

Table S1 **Clinical information of patients involved in study.** All cases were reviewed by pathologists to confirm HCC. HCC differentiation grade, Clinical stage-T, Clinical stage-N, Clinical stage-M, Clinical stage(BCLC system), status of HBsAg, HBcAg, date of First surgery, date of death are listed in the table.
(XLSX)

Table S2 **SNP sites of 10 oncogenes checked for HCC samples.** Mutation sites with the highest frequencies were selected according to the COSMIC website for HCC samples (http://www.sanger.ac.uk/genetics/CGP/cosmic/). Single-nucleotide polymorphisms (SNPs) in the CSF1R, CTNNB1, KRAS, BRAF, NRAS, ERBB2, MET, PIK3CA, JAK1, and SMO genes with frequencies higher than 0.1% mutation in HCC were listed in the table and checked in the study.
(DOC)

Table S3 **List of genes with transcription significantly altered.** Four paired samples derived from HBV-positive patients that harbored the CTNNB1 mutation were selected for RNA-seq. RNA single-end sequencing was performed using an Illumina GAII analyzer. 45-bp sequences called by the Illumina pipeline were mapped to the human genome (hg19). Gene annotation and the calculation of FPKM (fragments per kilobase of transcript per million mapped reads) values were determined. Differences in gene expression were assessed using Cuffdiff, with a false discovery rate correction for multiple testing. Genes with p-values ≤0.05 and a fold change ≥2 were considered differentially expressed. Significantly upregulated genes are listed in worksheet “up genes”; Significantly downregulated genes are listed in worksheet “down genes”.
(XLS)
