# Supplementary figures and images for: Transcriptomic Characterization of Hepatocellular Carcinoma with CTNNB1 Mutation
Source: PLoS One. 2014 May 5;9(5):e95307. doi: 10.1371/journal.pone.0095307 (PMC4010419; doi:10.1371/journal.pone.0095307)

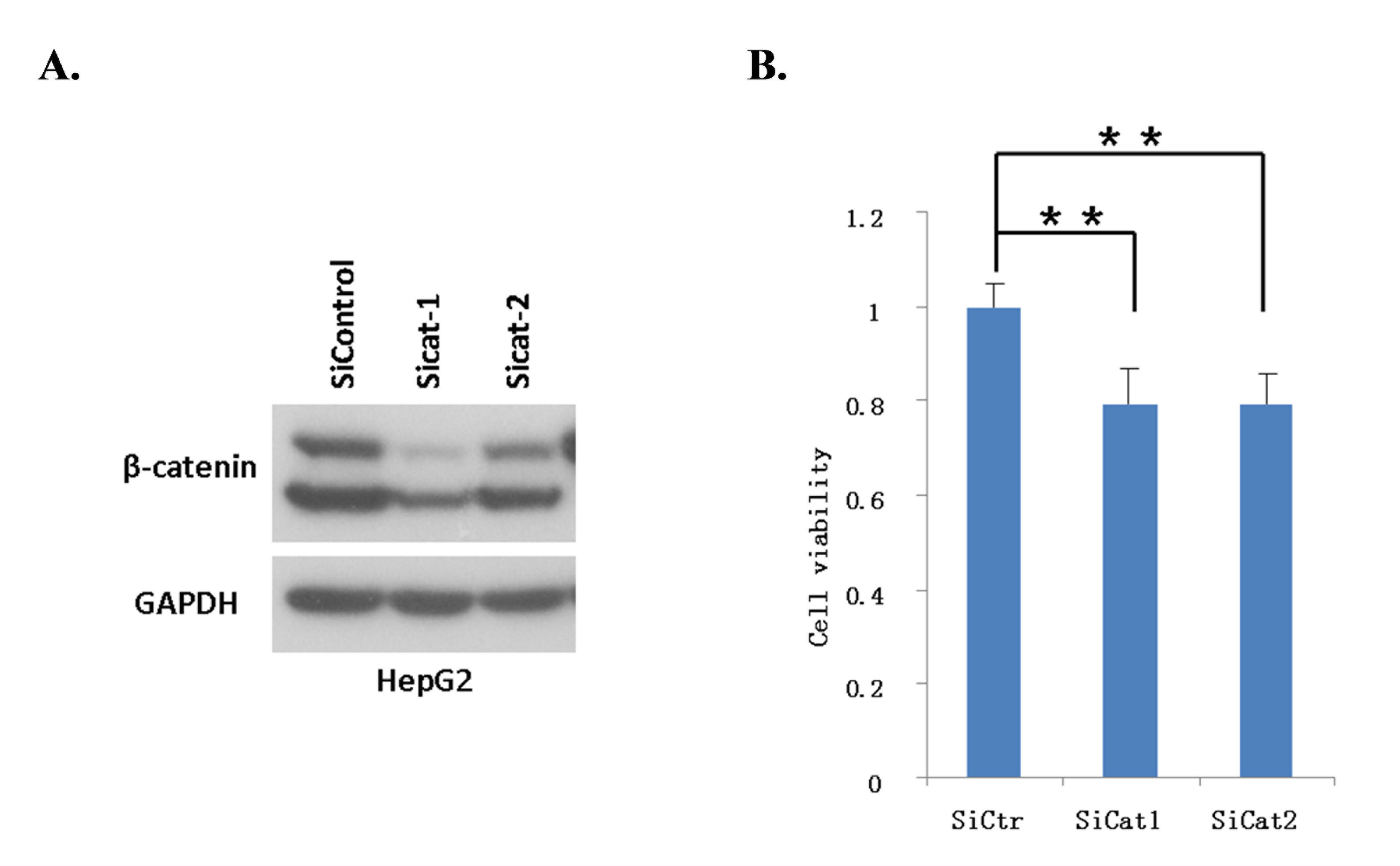

Supplement: Figure S1 — knockdown of β-catenin reduced growth of Hep2G cell line. A: Two siRNAs effectively knocked down protein level ofβ-catenin in HepG2 cell. B: β-catenin knocked down HepG2 cell showed significantly lower growth rate in comparison to that treated with control siRNA. HepG2 cells were transfected with human β-catenin (CTNNB1) siRNA or negative control siRNA. The cells were harvested at 48 and 72 hours post-transfection for western blotting and cell viability assay respectively. The siRNAs sequences used are listed below. siCat1: CCACAAGAUUACAAGAAACGGCUUU; siCat2: AAGUCCUGUAUGAGUGGGAAC; siCtr: AACAGUCGCGUUUGCGACUGG. (TIF) [file pone.0095307.s001.tif]
