# Supplementary material for: Transcriptomic Characterization of Hepatocellular Carcinoma with CTNNB1 Mutation
Source: PLoS One. 2014 May 5;9(5):e95307. doi: 10.1371/journal.pone.0095307 (PMC4010419; doi:10.1371/journal.pone.0095307)
Supplement: Table S2 — SNP sites of 10 oncogenes checked for HCC samples. Mutation sites with the highest frequencies were selected according to the COSMIC website for HCC samples (http://www.sanger.ac.uk/genetics/CGP/cosmic/). Single-nucleotide polymorphisms (SNPs) in the CSF1R, CTNNB1, KRAS, BRAF, NRAS, ERBB2, MET, PIK3CA, JAK1, and SMO genes with frequencies higher than 0.1% mutation in HCC were listed in the table and checked in the study. (DOC) [file pone.0095307.s003.doc]

| oncogene | SNP sites | amino acids change | mutation frequency |
| --- | --- | --- | --- |
| CSF1R | 902T>C | L301S | 3/14 |
| KRAS | 34G>T | G12C | 3/360 |
| KRAS | 35G>A | G12D | 8/360 |
| KRAS | 35G>T | G12V | 5/360 |
| KRAS | 37G>T | G13C | 1/360 |
| KRAS | 38_39GC>AT | G13D | 1/360 |
| KRAS | 38G>A | G13D | 1/360 |
| KRAS | 183A>T | Q61H | 3/360 |
| BRAF | 1799T>A | V600E | 2/57 |
| NRAS | 181C>A | Q61K | 3/222 |
| NRAS | 182A>T | Q61L | 7/222 |
| ERBB2 | 2632C>T | H878Y | 2/152 |
| MET | 3572C>T | T1191I | 1/64 |
| MET | 3785A>G | K1262R | 1/64 |
| MET | 3804G>A | M1268I | 1/64 |
| PIK3CA | 398A>T | D133V | 1/352 |
| PIK3CA | 1634A>C | E545A | 1/352 |
| PIK3CA | 3101A>G | E1034G | 1/352 |
| PIK3CA | 3140A>G | H1047R | 2/352 |
| PIK3CA | 3140A>T | H1047L | 1/352 |
| PIK3CA | 3204_3205insA | N1068fs*4 | 13/352 |
| JAK1 | 1932G>T | Q644H | 1/178 |
| JAK1 | 1933G>T | V645F | 1/178 |
| SMO | 1724A>T | K575M | 1/26 |
| CTNNB1 | 94G>T/A | D32N | 39/3590 |
| CTNNB1 | 95A>G | D32G | 19/3590 |
| CTNNB1 | 98C>G | S33C | 29/3590 |
| CTNNB1 | 100G>A | G34R | 19/3590 |
| CTNNB1 | 101G>A/T | G34E/V | 42/3590 |
| CTNNB1 | 110C>G/T | D37C/F | 36/3590 |
| CTNNB1 | 121A>G | T41A | 56/3590 |
| CTNNB1 | 133T>C | S45P | 40/3590 |
| CTNNB1 | 134C>T | S45F | 35/3590 |

Supplementary Table 1: SNP sites of 10 oncogenes
